# A procedure for the preparation and isolation of nucleoside-5’-diphosphates

**DOI:** 10.3762/bjoc.11.52

**Published:** 2015-04-10

**Authors:** Heidi J Korhonen, Hannah L Bolt, David R W Hodgson

**Affiliations:** 1 Department of Chemistry, Science Laboratories, Durham University, South Road, Durham, DH1 3LE, United Kingdom,; 2 Department of Chemistry, University of Turku, Vatselankatu 2, 20014 Turku, Finland

**Keywords:** NDP synthesis, nucleic acids, nucleoside-5’-diphosphate, phosphorylation

## Abstract

Tris[bis(triphenylphosphoranylidene)ammonium] pyrophosphate (PPN pyrophosphate) was used in the S_N_2 displacements of the tosylate ion from 5’-tosylnucleosides to afford nucleoside-5’-diphosphates. Selective precipitation permitted the direct isolation of nucleoside-5’-diphosphates from crude reaction mixtures.

## Introduction

Nucleoside-5'-phosphates are key to many mechanistic studies and chemical biology applications [1–3]. Synthetic approaches towards nucleoside-5'-phosphates are well-established [4], however, the methods tend to be cumbersome. Newer approaches have started to become available over the last decade, where a key driver in these approaches is limiting the effects of moisture on the reaction outcome [5–11].

Nucleoside-5'-triphosphates (NTPs) are frequently accessed via the Ludwig modification [12] of the Yoshikawa procedure [13–14], where an unprotected ribonucleoside is first phosphophorylated, then the resulting phosphodichloridate is captured using pyrophosphate ions. Unfortunately, similar attempts towards nucleoside-5'-diphosphates (NDPs), with phosphate ion as nucleophile, tend to produce greater quantities of NTP than NDP [15–16]. Nuceloside-5’-diphosphates can, however, be accessed via S_N_2 displacement at 5’-activated nucleosides using pyrophosphate as the nucleophile. This approach, developed by Poulter and co-workers [17], forms a cornerstone in the armoury towards the synthesis of nucleoside-5’-polyphosphates [4]. The key element to the success of this process is the availability of dry, organic-solvent-soluble pyrophosphate. Tris(tetrabutylammonium) pyrophosphate is generally employed in this role [18], however, isolating this material in a dry condition, and maintaining its dryness are significant problems [4,19]. In addition, excess pyrophosphate is usually employed in order to drive the kinetics of the displacement process, however, this excess material tends to co-elute with nucleoside-5’-diphosphate products during anion exchange chromatographic purifications.

**Scheme 1:**
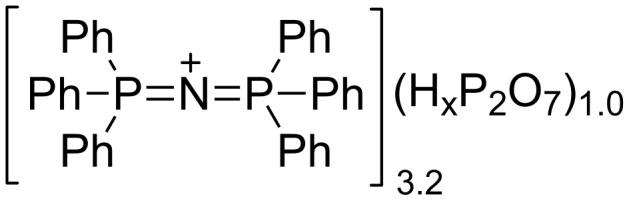
PPN pyrophosphate.

We have recently reported the successful synthesis of PPN pyrophosphate (Scheme 1) and its application in the syntheses of nucleoside-5’-triphosphates (NTPs) [11]. PPN pyrophosphate is straightforward to prepare, and, unlike alkylammonium salts, shows limited levels of moisture uptake. In this letter we explore the use of PPN pyrophosphate as a replacement for tris(tetrabutylammonium) pyrophosphate in the preparation of NDPs via the Poulter approach [17]. Thereafter, we consider the removal of the PPN cation from reaction mixtures alongside removal of excess pyrophosphate to facilitate the isolation of NDP products.

## Results and Discussion

**Scheme 2:**
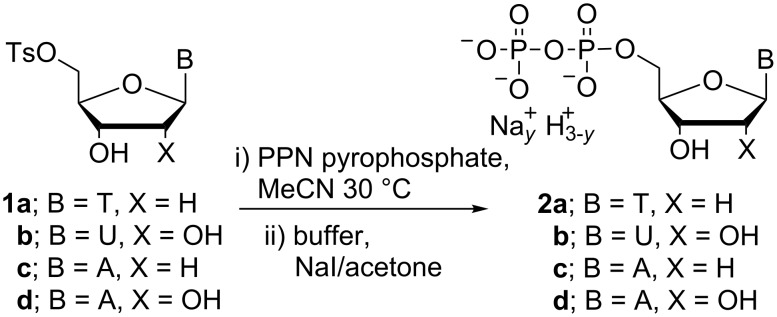
Preparation of NDPs.

Preliminary experiments focused on 5’-tosylthymidine **1a** as a test substrate (Scheme 2), using conditions similar to those employed by Poulter and co-workers [17]. An excess of pyrophosphate was used to drive the kinetics of the displacement process in concentrated solutions in acetonitrile as the reaction solvent. The reaction progress was monitored via ^31^P NMR spectroscopy, and ~62% conversion to TDP **2a** was attained after 30 h of reaction based on pyrophosphate consumption. We found that slightly elevated temperature (30 °C) accelerated the displacement, with no adverse effect on conversion levels to NDP. We believe that our use of PPN pyrophosphate supports the use of elevated temperatures because it limits (or even eliminates) the possibility of water ingress into reaction mixtures and the resulting hydrolysis processes that lead to the formation of monophosphate byproducts. Additional 5’-tosylnucleosides **1b–d** were then exposed to the same reaction conditions and reaction progress was monitored by ^31^P NMR spectroscopy (Table 1).

**Table 1:** Reaction times, yields and purities of 5’-diphosphates.

Nucleoside	Time [h]	Yield [%]	Purity [%]^a^	Product

**1a**	30	45	98	**2a**
**1b**	91	40	98	**2b**
**1c**	91	44	92	**2c**
**1d**	91	20	87	**2d**

^a^Determined by ^31^P NMR spectroscopy.

**Scheme 3:**
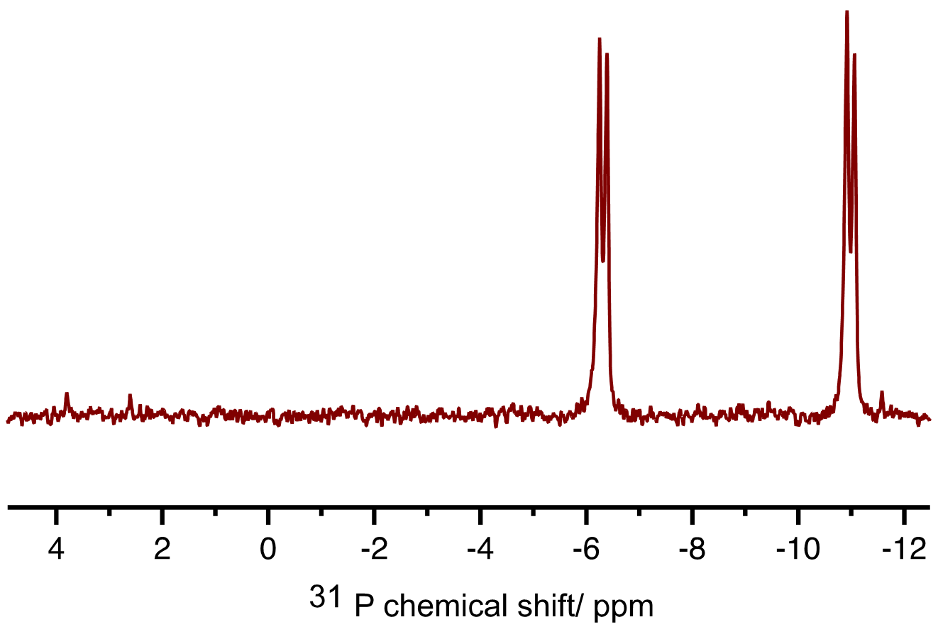
^31^P NMR spectrum of TDP **2a** after precipitation from reaction mixture.

In order to remove inorganic pyrophosphate from the crude reaction mixtures we developed selective precipitation procedures. To ensure that these procedures were readily repeatable, we used triethylammonium bicarbonate (TEAB) buffer to control the pH value, and we used ice baths to ensure constant conditions of temperature. Owing to its charge-dense nature, we hoped to be able to precipitate pyrophosphate as its sodium salt through the use of sodium iodide solution in acetone, where acetone served as the precipitating solvent. A systematic study of conditions was performed in order to allow selective precipitation of pyrophosphate ions. Variables included; TEAB buffer concentration and volume, the concentration of the sodium iodide solution in acetone and the volume of this solution that was added. The effectiveness of conditions was assessed using ^31^P NMR spectroscopic analyses of both precipitated and supernatant materials, and, conveniently, we found conditions that removed the pyrophosphate effectively. Thereafter, NDPs were isolated through the addition of further volumes of sodium iodide solution in acetone. Gratifyingly, the sequence of selective precipitations allowed for the removal of both pyrophosphate and PPN ions, and isolated materials showed high levels of purity, and reasonable levels of mass recovery without chromatographic purifications (Scheme 3, Table 1).

**Scheme 4:**
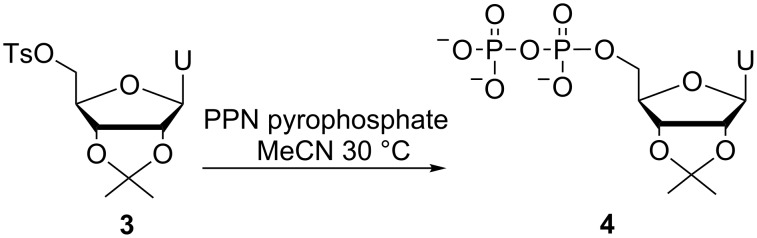
Attempted use of an isopropylidene-protected 5’-tosylnucleoside.

Protected substrate **3** showed conversion to diphosphate **4** after 91 h of reaction, however, we were unable to precipitate this material after removal of excess pyrophosphate ions (Scheme 4). We believe the isopropylidene protecting group decreases the polarity of diphosphate **4** to such an extent that it remains soluble even after the addition of excess sodium iodide and acetone.

## Conclusion

PPN pyrophosphate is a convenient, effective reagent for the preparation of NDPs from 5’-tosylnucleosides. Post-reaction removal of PPN cations and elimination of excess pyrophosphate followed by isolation of polar NDPs as sodium salts can be readily achieved through selective precipitation.

## Experimental

### General procedure for the synthesis of NDPs **2a–d**

PPN pyrophosphate was prepared as described previously [11] and was dried in a vacuum desiccator over P_2_O_5_ (we have also found freeze-drying to be effective in other experiments), then dissolved in dry acetonitrile, and stored over activated 4 Å molecular sieves. Tosylated nucleoside **1a–d** (0.18 mmol) and PPN pyrophosphate (0.36 mmol) in dry acetonitrile (0.8 mL) were stirred at 30 °C under an inert atmosphere. The reaction progress was monitored by ^31^P NMR spectroscopy until no further reaction was observed. Sodium iodide (0.149 g) was dissolved into 9:1 acetone/water (10 mL) and the solution was added dropwise with stirring to the crude reaction mixture which had been transferred to a centrifuge tube. The solution was mixed for 30 minutes at 0 °C and then centrifuged at 4,000 rpm for 10 minutes. The supernatant was removed by a pipette and the solid residue was dissolved in TEAB buffer (3 mL, 0.1 M, pH 8.0) [4]. Sodium iodide in acetone (5 mL, 0.1 M) was added slowly to the solution and the mixture was stirred for 30 minutes at 0 °C. Solids were collected by centrifugation at 4,000 rpm for 10 minutes, and the liquid layer was analysed by ^31^P NMR spectroscopy to ensure that selective removal of inorganic pyrophosphate had been achieved. In cases where pyrophosphate ions were still present, an additional small volume (<1 mL) of sodium iodide in acetone was added and the mixture was stirred and solids were collected by centrifugation as described above. When all pyrophosphate had been removed from the supernatant layer (determined by ^31^P NMR spectroscopy), sodium iodide in acetone (5 mL, 0.1 M) was added in order to precipitate the NDP product. The mixture was stirred for 30 minutes at 0 °C and then centrifuged at 4,000 rpm for 10 minutes. The precipitated NDP was washed with acetone (2 × 2 mL) in order to remove remaining sodium iodide and dried in a vacuum desiccator to yield a white solid.

## Supporting Information

File 1 Experimental procedures for the preparation of 5’-tosylates and their ^1^H and ^13^C NMR spectra, and ^31^P and ^1^H NMR spectra of NDPs.
