# Supplementary material for: A procedure for the preparation and isolation of nucleoside-5’-diphosphates
Source: Beilstein J Org Chem. 2015 Apr 10;11:469–72. doi: 10.3762/bjoc.11.52 (PMC4419508; doi:10.3762/bjoc.11.52)
Supplement: File 1 — Experimental procedures for the preparation of 5’-tosylates and their 1H and 13C NMR spectra, and 31P and 1H NMR spectra of NDPs. [file Beilstein_J_Org_Chem-11-469-s001.pdf]

Supporting Information

for

A Procedure for the Preparation and Isolation of Nucleoside-5’-Diphosphates

Heidi J. Korhonen[1,2], Hannah L. Bolt[1] and David R. W. Hodgson*[1]

Address: [1]Department of Chemistry, Science Laboratories, Durham University, South Road, Durham, DH1 3LE, United Kingdom, and [2]Department of Chemistry, Vatselankatu 2, 20014, University of Turku, Finland.

Email: David R. W. Hodgson - d.r.w.hodgson@durham.ac.uk

*Corresponding author

Experimental procedures for the preparation of 5’-tosylates and their ^{1}H and ^{13}C NMR spectra, and ^{31}P and ^{1}H NMR spectra of NDPs

Contents

The Preparation of 5’-Tosylates s3

^{1}H NMR of 5’-*O*-tosylthymidine (**1a**) s4

^{13}C NMR of 5’-*O*-tosylthymidine (**1a**) s5

^{1}H NMR of 5’-*O*-tosyluridine (**1b**) s6

^{13}C NMR of 5’-*O*-tosyluridine (**1b**) s7

^{1}H NMR of 2’-deoxy-5’-*O*-tosyladenosine (**1c**) s8

^{13}C NMR of 2’-deoxy-5’-*O*-tosyladenosine (**1c**) s9

^{1}H NMR of 2’,3’-*O*-isopropylidene-5’-*O*-tosyluridine (**3**) s10

^{13}C NMR of 2’,3’-*O*-isopropylidene-5’-*O*-tosyluridine (**3**) s11

^{31}P NMR of crude TDP reaction mixture s12

^{31}P NMR of thymidine 5´-diphosphate (**2a**) s13

^{1}H NMR of thymidine 5´-diphosphate (**2a**) s14

^{31}P NMR of uridine 5´-diphosphate (**2b**) s15

^{1}H NMR of uridine 5´-diphosphate (**2b**) s16

^{31}P NMR of 2´-deoxyadenosine 5´-diphosphate (**2c**) s17

^{1}H NMR of 2´-deoxyadenosine 5´-diphosphate (**2c**) s18

^{31}P NMR of adenosine 5´-diphosphate (**2d**) s19

^{1}H NMR of adenosine 5´-diphosphate (**2d**) s20

References s21

The Preparation of 5'-Tosylates

2′,3′-*O*-Isopropylideneuridine and 5'-tosyladenosine **1d** were purchased from Carbosynth and used as received. 5´-tosylthymidine was synthesized according to a literature procedure [1] to give **1a** as a white powder (1.70 g, 51%). 2'-Deoxy-5'-tosyladenosine **1c** was prepared from 2'-deoxyadenosine hydrate via a published procedure [2] to yield 1.97 g (60%) of **1c**.

2',3'-O-Isopropylidene-5'-O-tosyluridine **3**: p-toluenesulfonyl chloride (1.34 g, 7.00 mmol) was dissolved in pyridine (5 ml) and added dropwise to a solution of 2´,3´-*O*-isopropylideneuridine (1.60 g, 5.63 mmol) in dry pyridine (10 ml) at 0 °C, and the mixture was stirred at 0 °C for 2 h. The mixture was added to an ice/water mixture (100 ml), stirred for 20 min. and extracted with ethyl acetate (2 × 120 ml). The organic layers were combined and washed with saturated sodium hydrogen carbonate solution (2 × 20 ml) and distilled water (2 × 20 ml), and dried over magnesium sulfate. The solution was filtered, the solvent removed *in vacuo*, and the residue was subjected to silica gel chromatography (CH_2Cl_3:MeOH, 50:1) to yield 1.62 g (65%) of 2´,3´-*O*-isopropylidene-5'-*O*-toluenesulfonyluridine. ^{1}H and ^{13}C NMR spectra correspond to data in the literature [3,4].

5'-O-Tosyluridine **1b**: 2',3'-*O*-isopropylidene-5'-*O*-toluenesulfonyluridine (0.740 g, 1.69 mmol) was stirred in 90% TFA in water (2 ml) for 10 min. The solvent was removed in vacuo and the product was isolated by silica gel chromatography (CH_2Cl_2:MeOH, 9:1) to yield 0.483 g (71%) of 5'-*O*-tosyluridine. ^{1}H and ^{13}C NMR spectra correspond to data in the literature [1].

^{1}H NMR of 5'-*O*-tosylthymidine (1a)

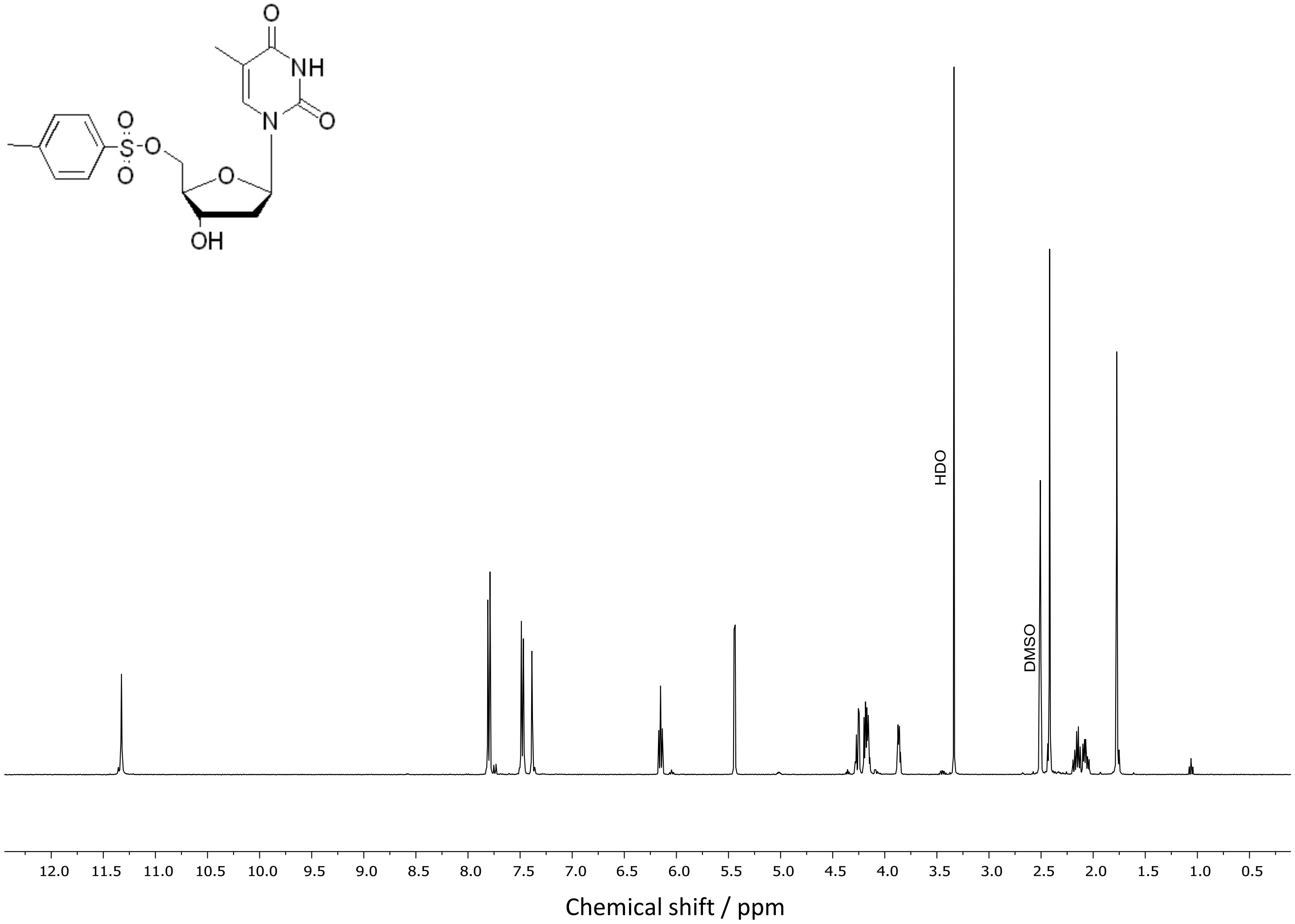

^{13}C NMR of 5’-*O*-tosylthymidine (1a)

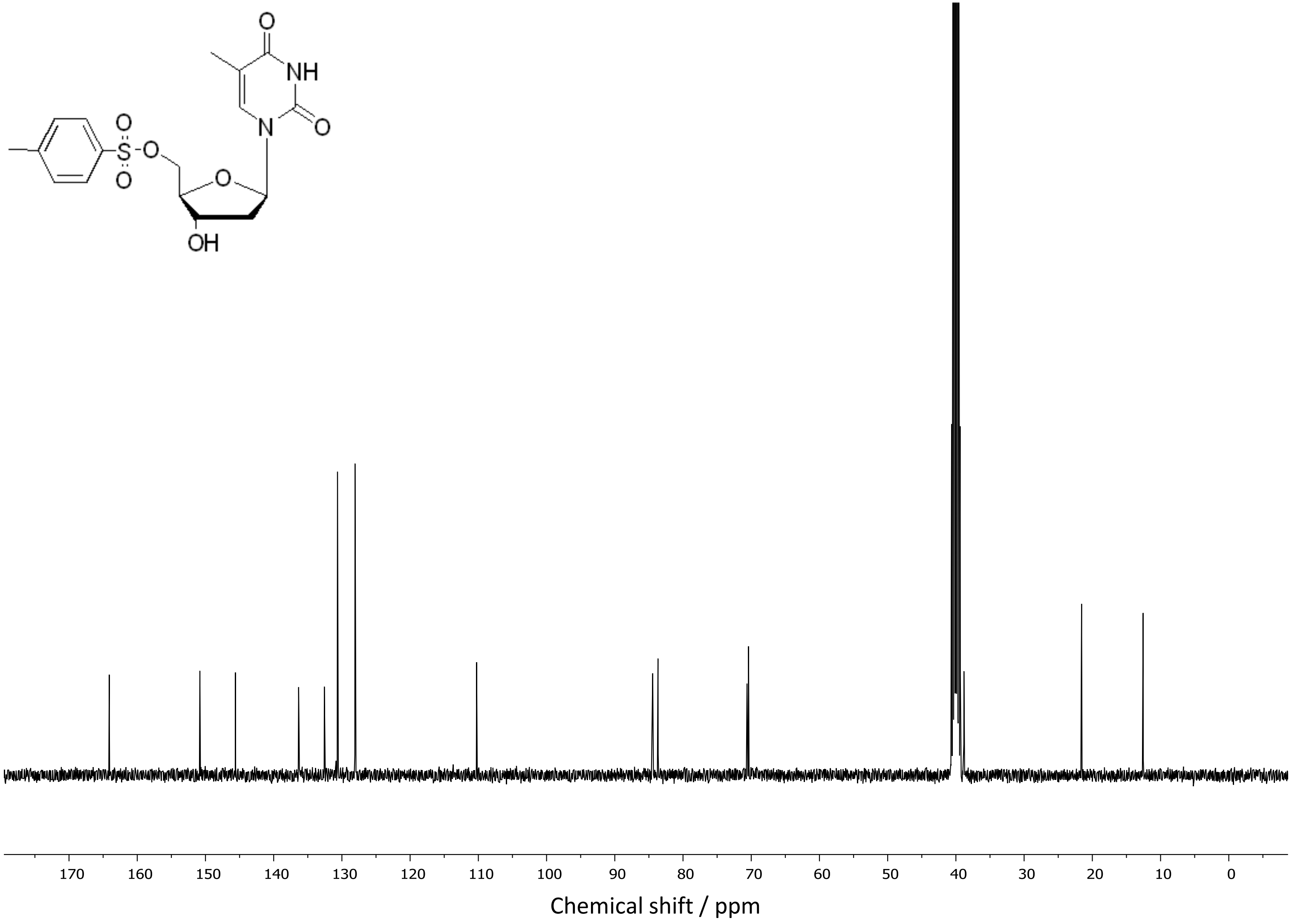

^{1}H NMR of 5'-*O*-tosyluridine (1b)

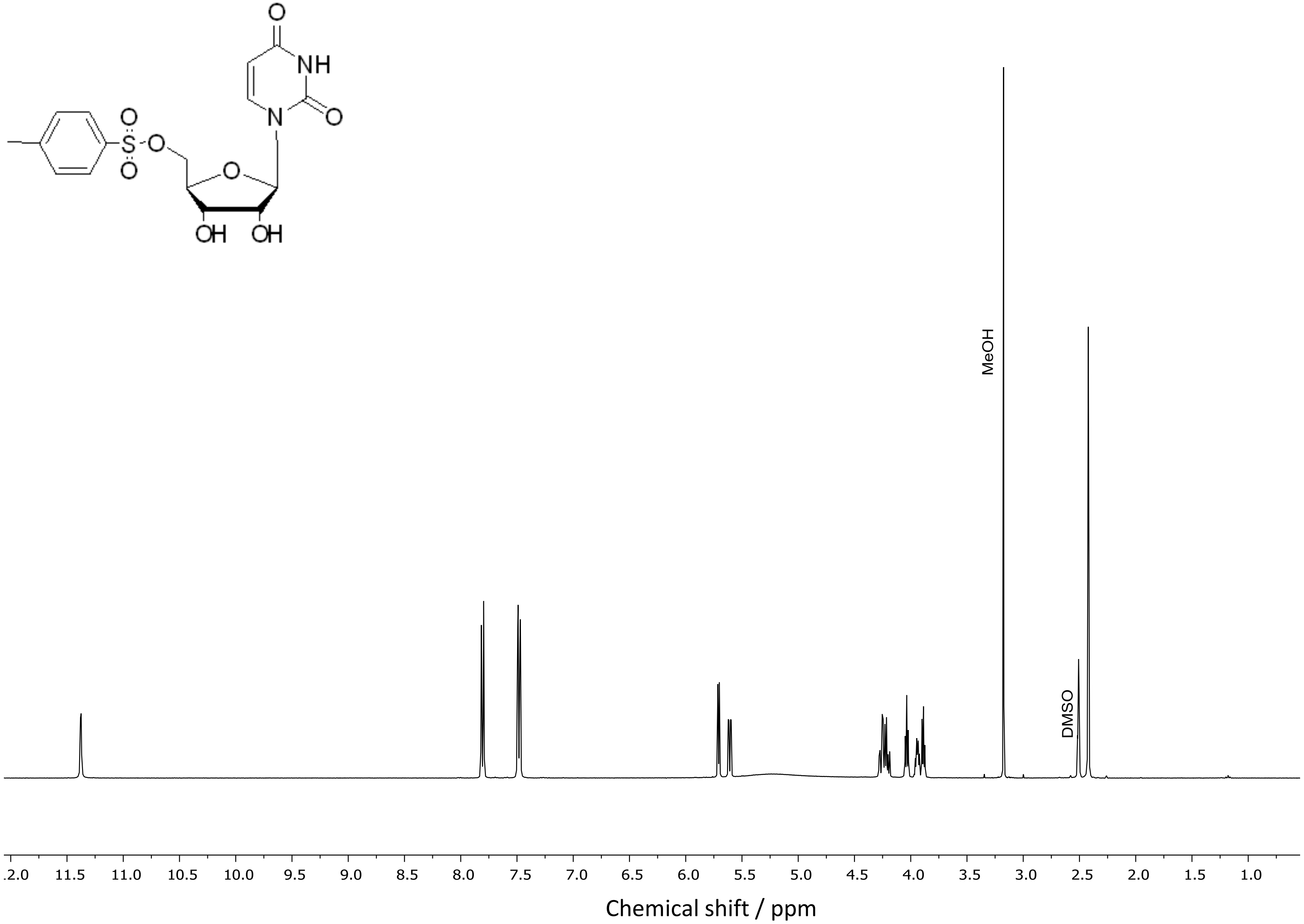

^{13}C NMR of 5'-*O*-tosyluridine (1b)

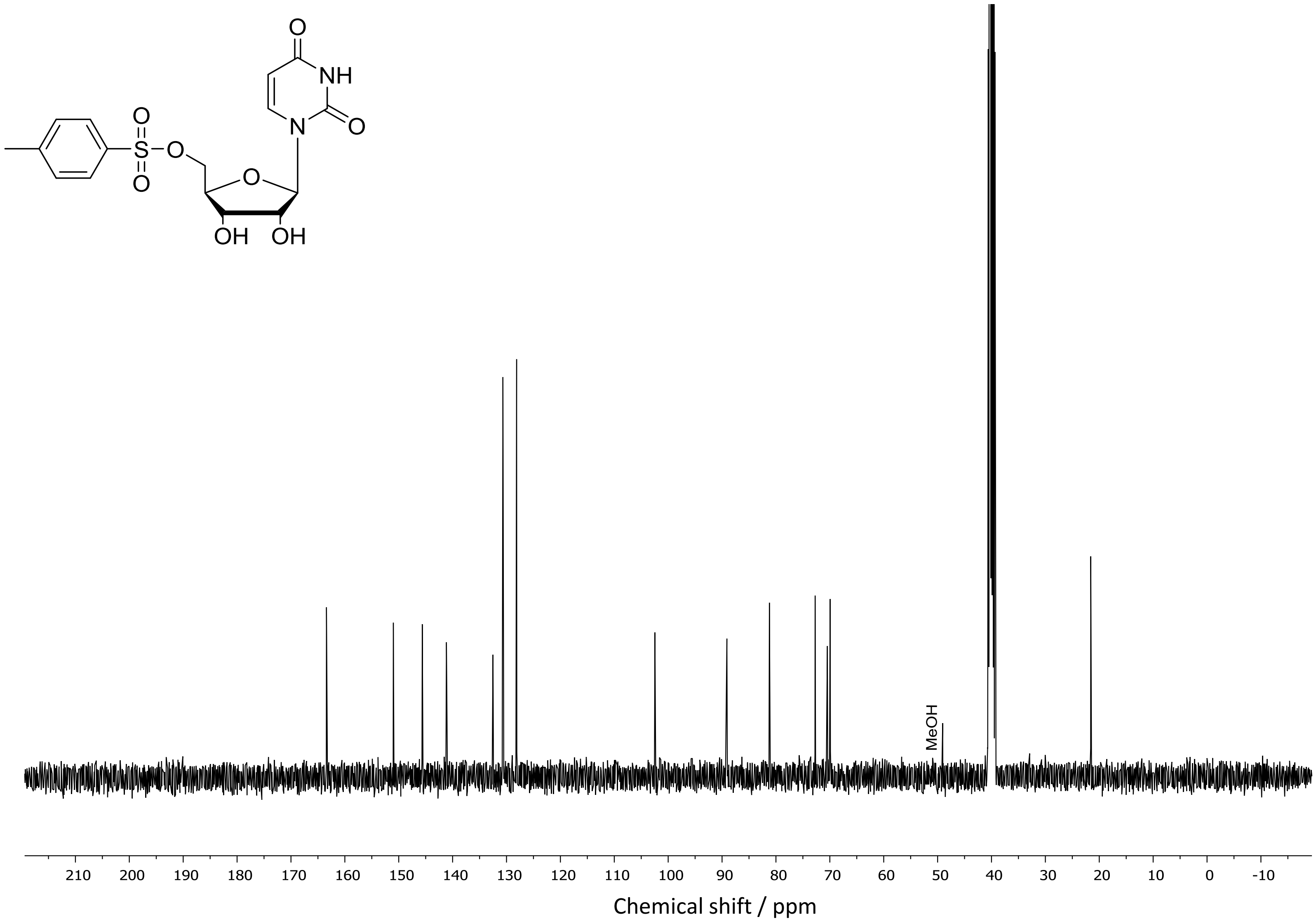

^{1}H NMR of 2’-deoxy-5’-*O*-tosyladenosine (1c)

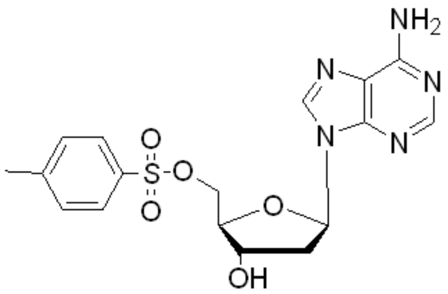

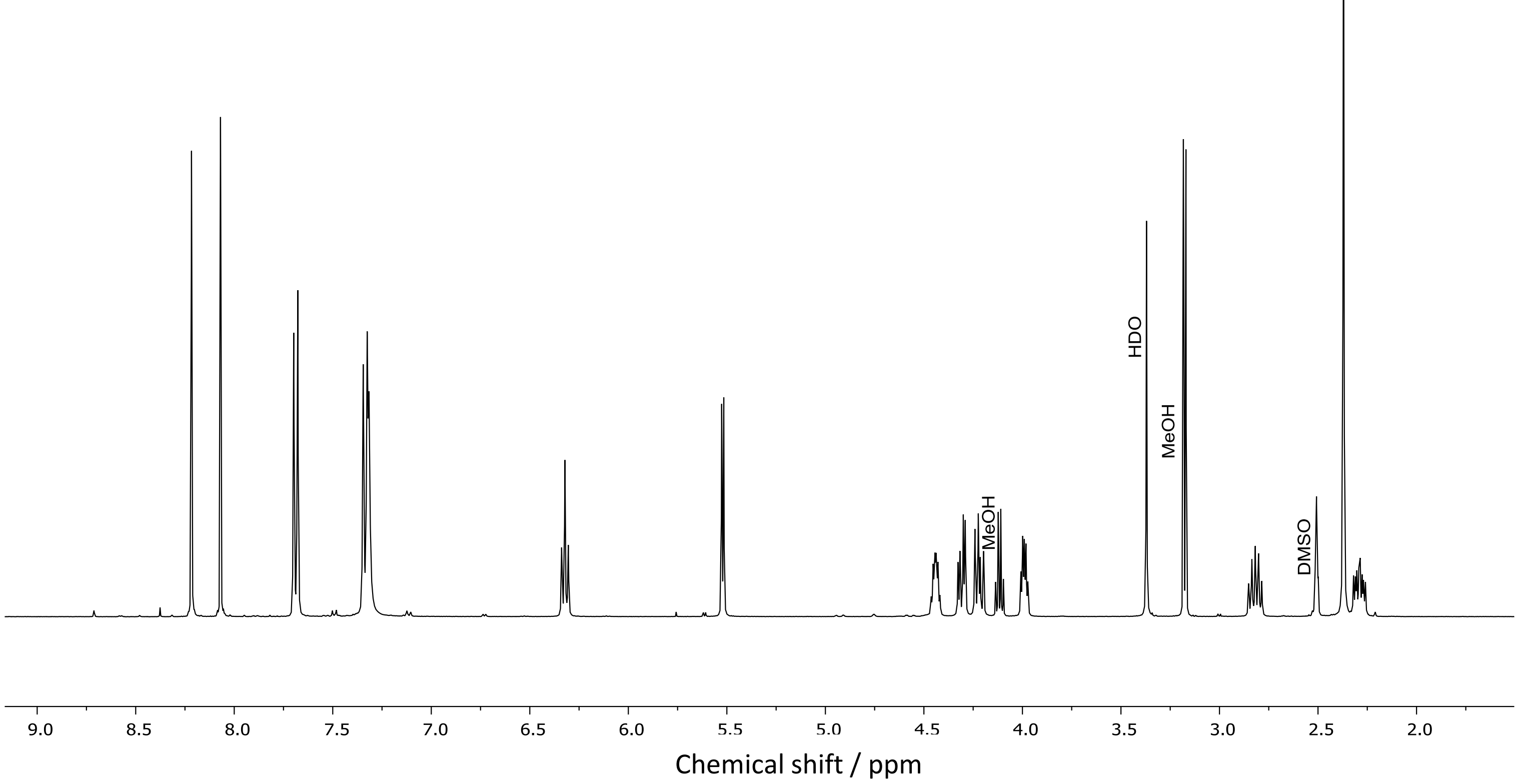

^{13}C NMR of 2'-deoxy-5'-*O*-tosyladenosine (1c)

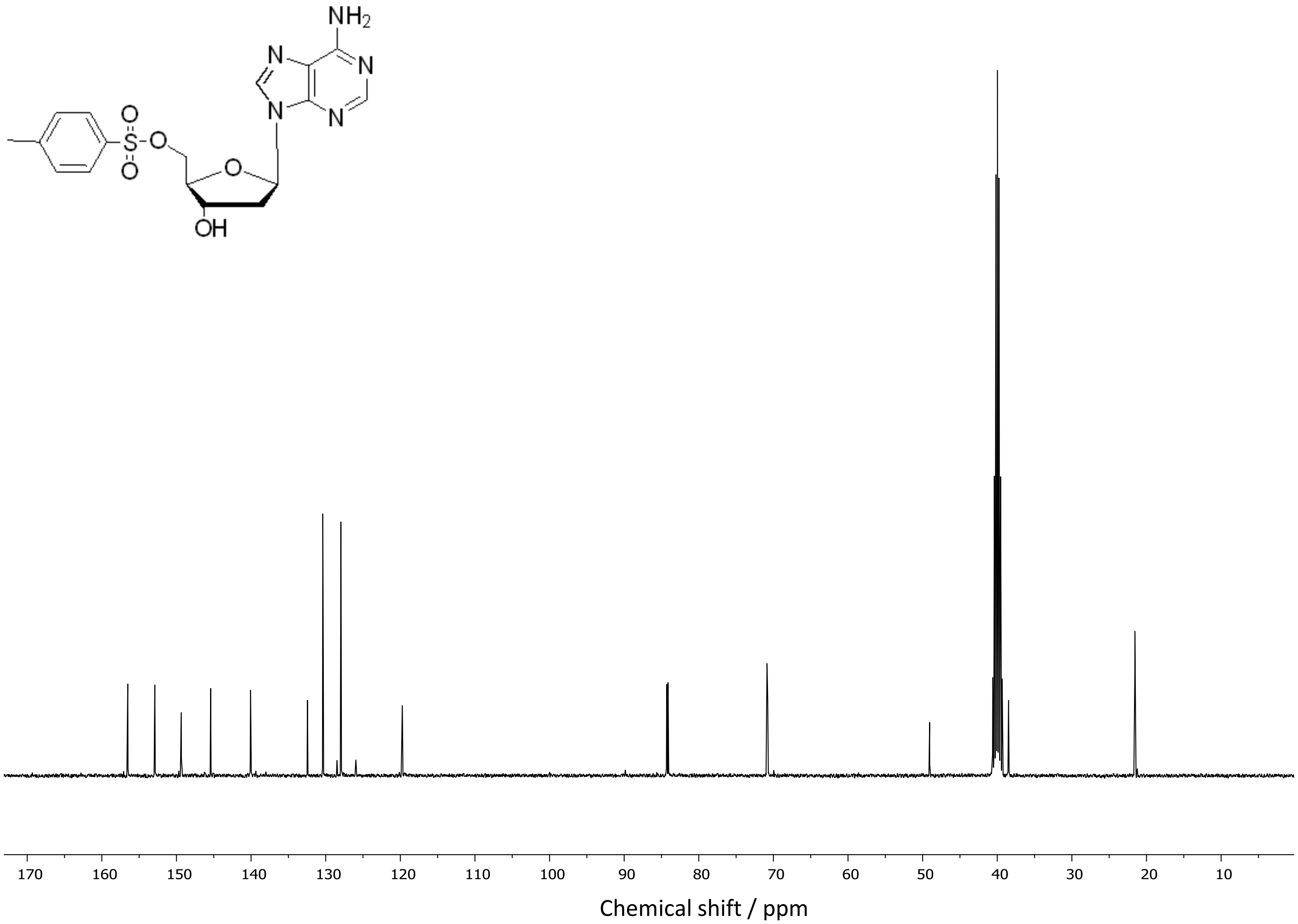

^{1}H NMR of 2',3'-*O*-isopropylidene-5'-*O*-tosyluridine (3)

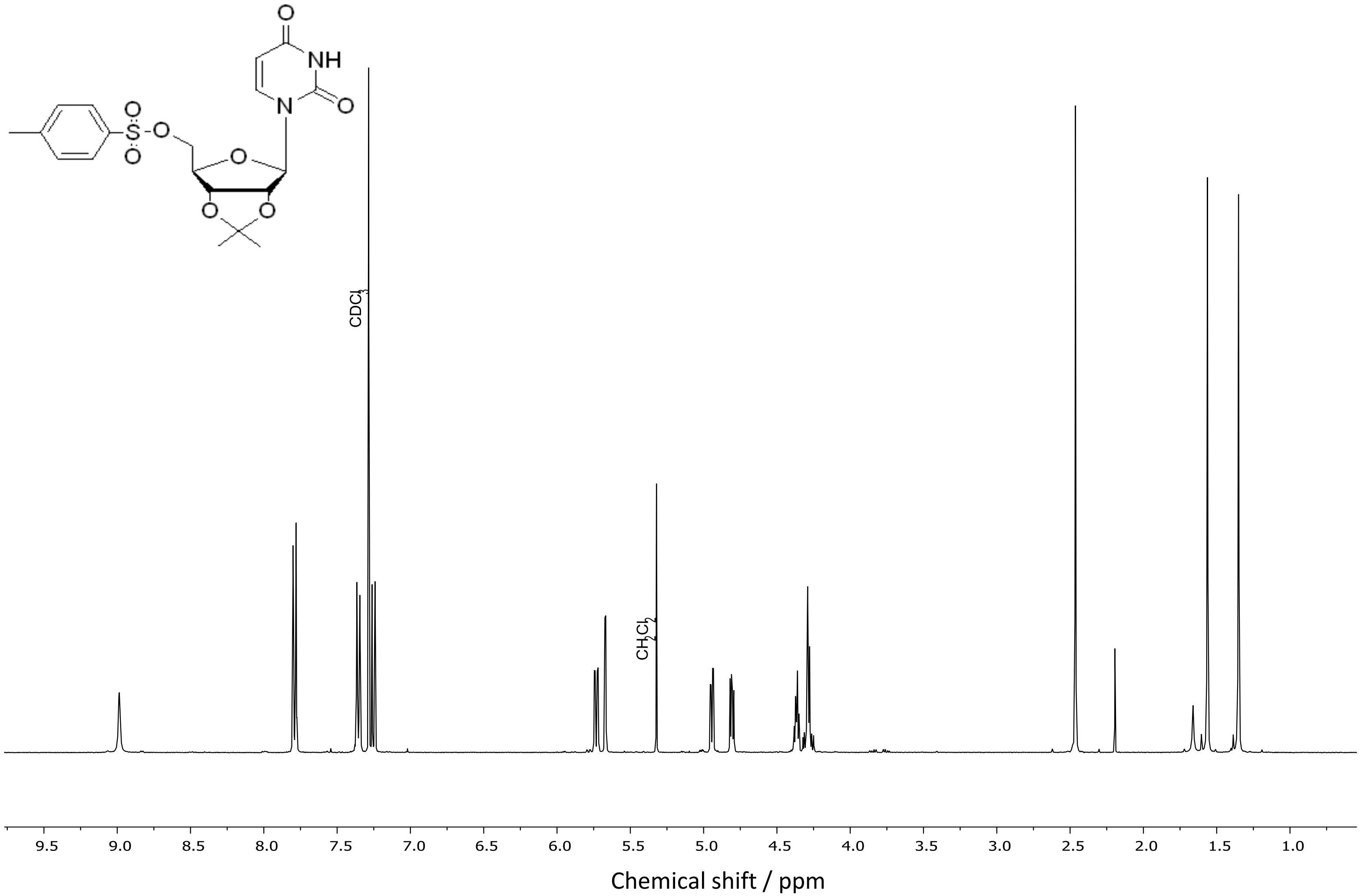

^{13}C NMR of 2',3'-*O*-isopropylidene-5'-*O*-tosyluridine (3)

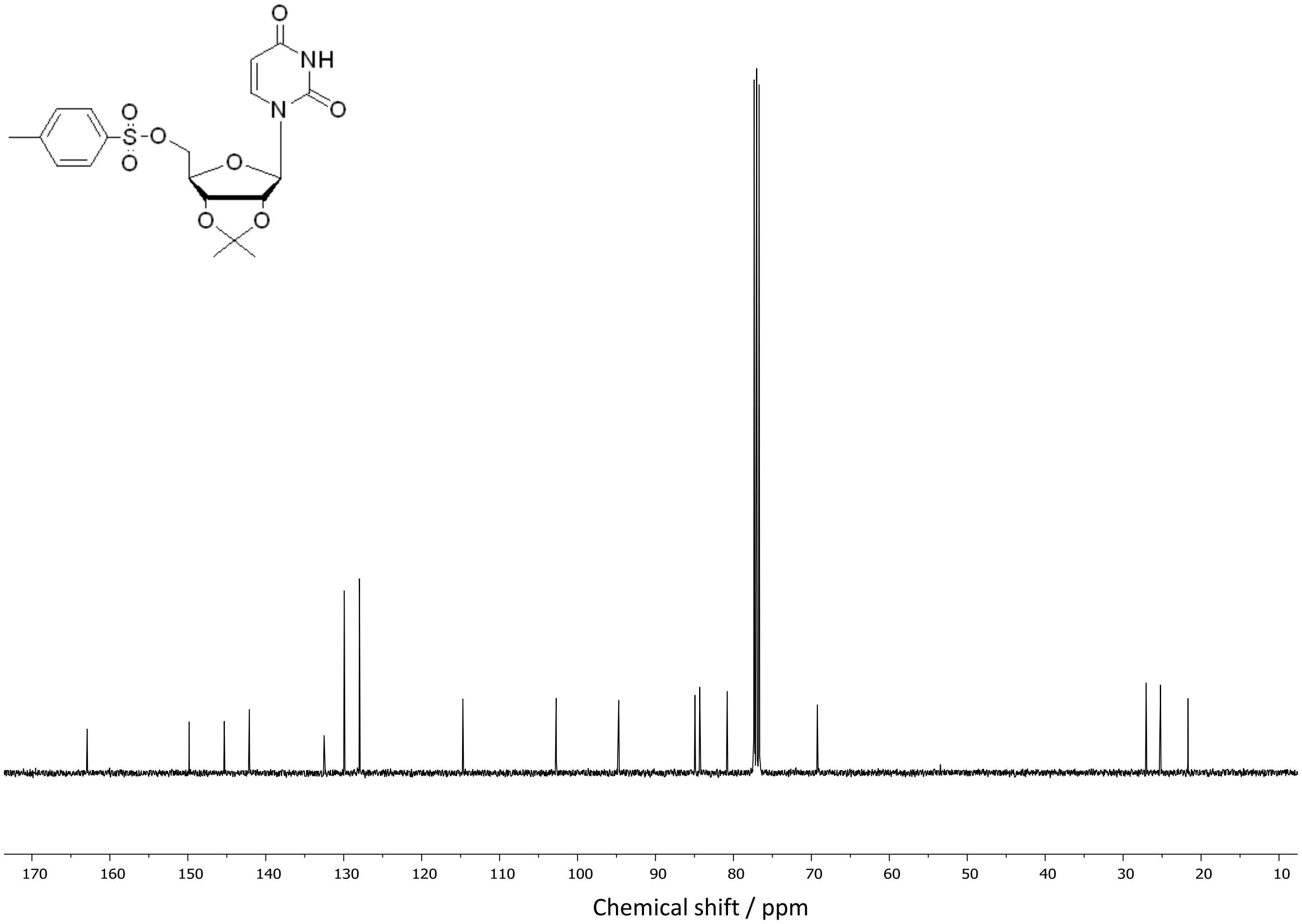

^{31}P NMR of crude TDP reaction mixture

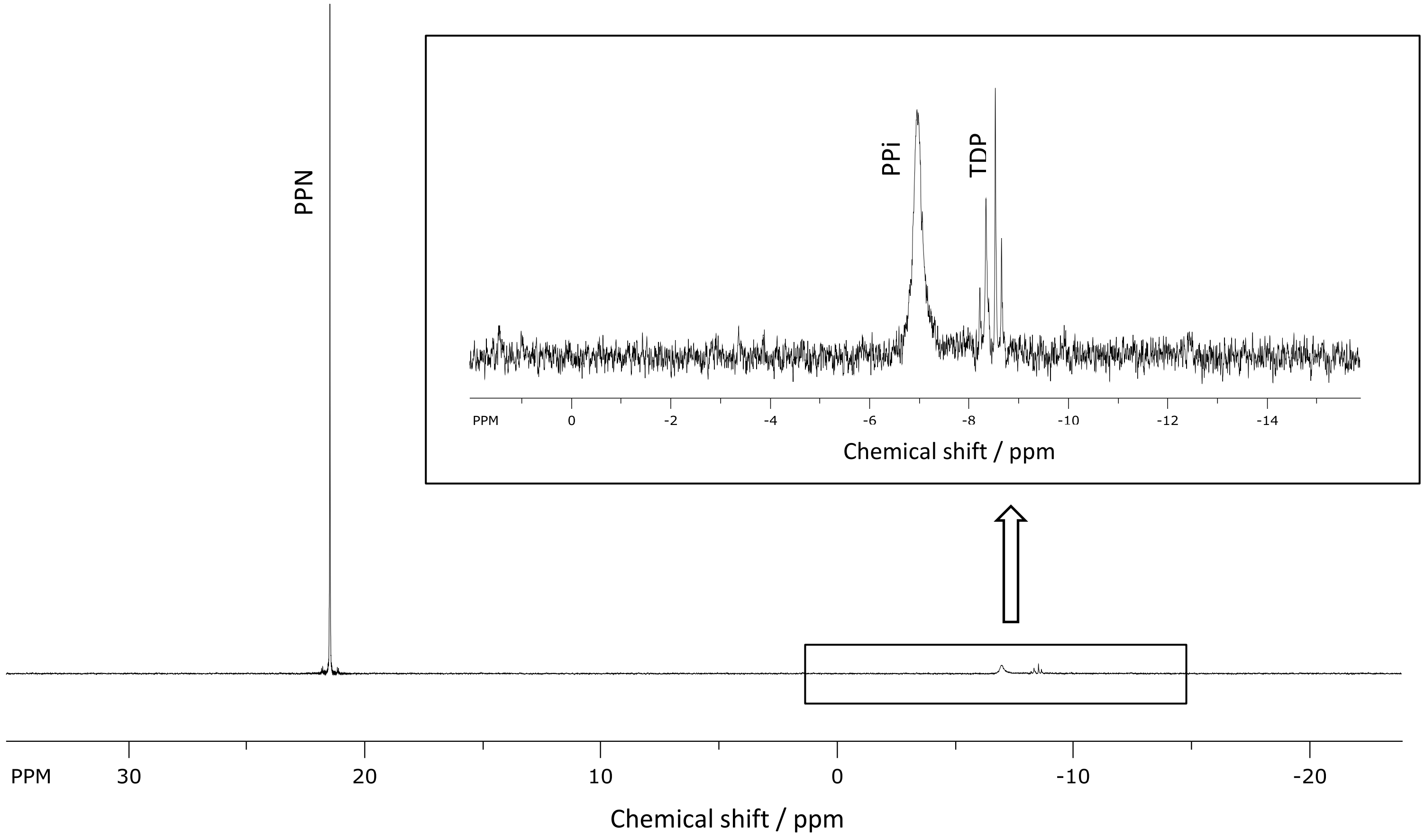

^{31}P NMR of thymidine 5´-diphosphate (2a)

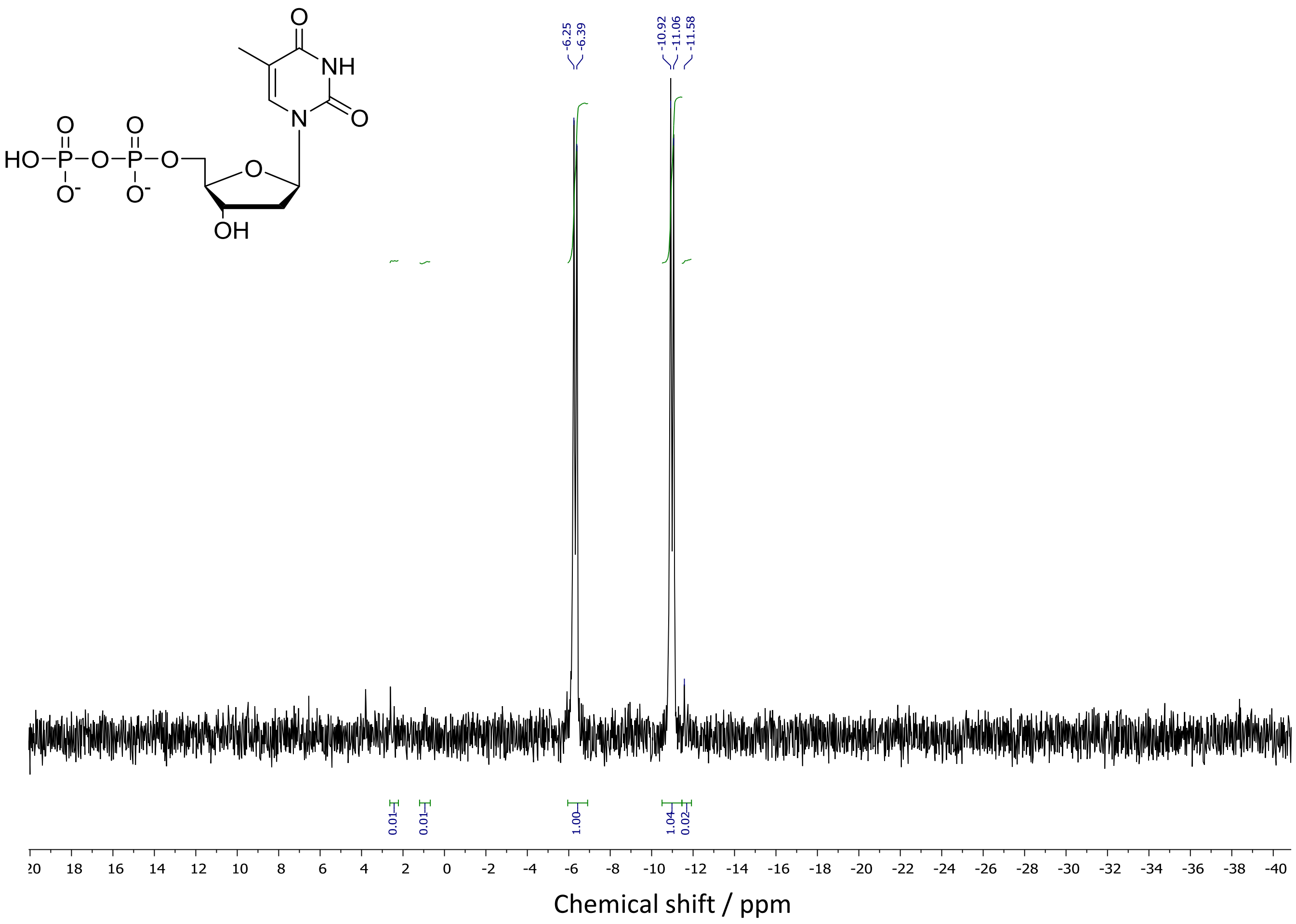

^{1}H NMR of thymidine 5´-diphosphate (2a)

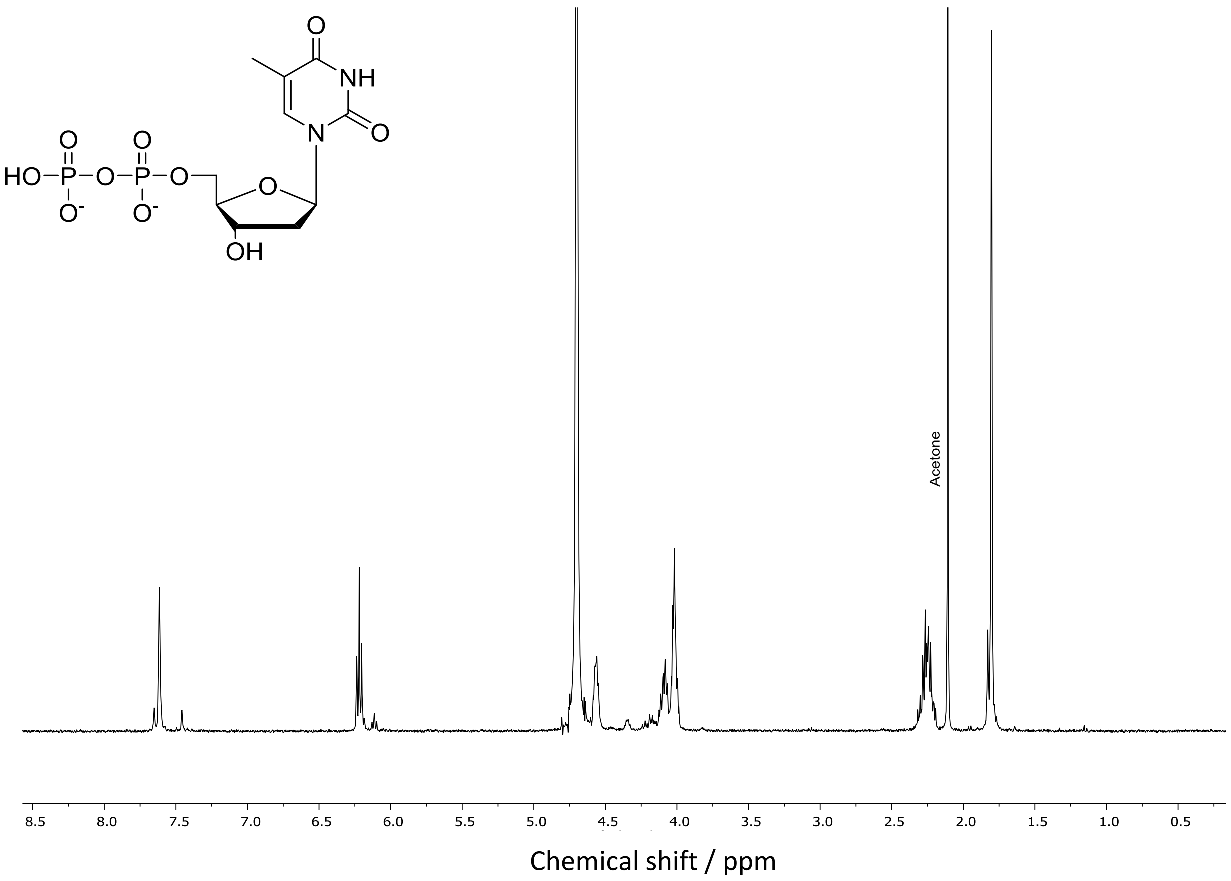

^{31}P NMR of uridine 5´-diphosphate (2b)

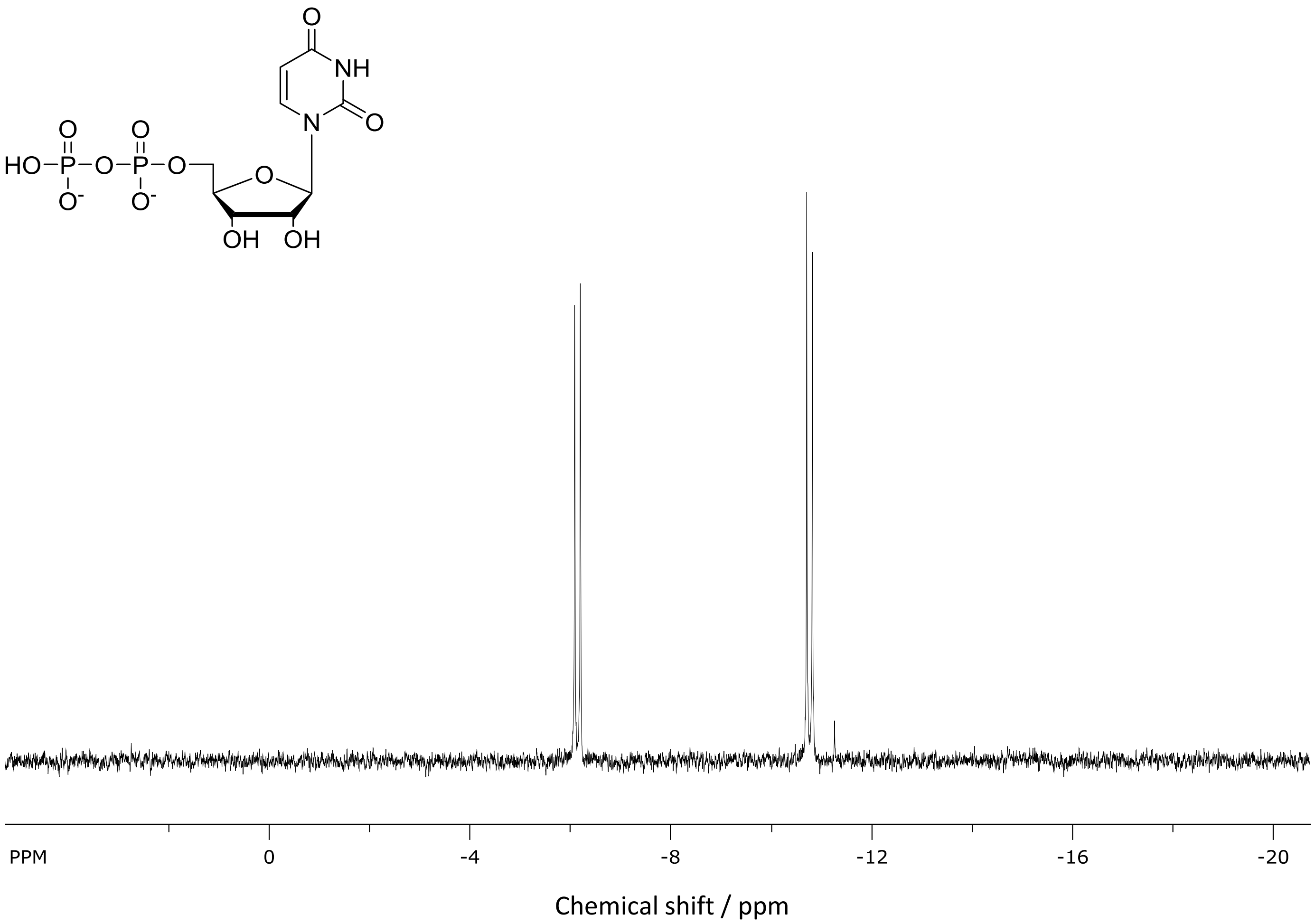

^{1}H NMR of uridine 5´-diphosphate (2b)

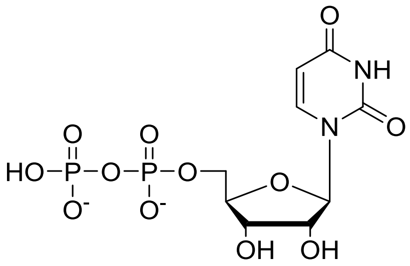

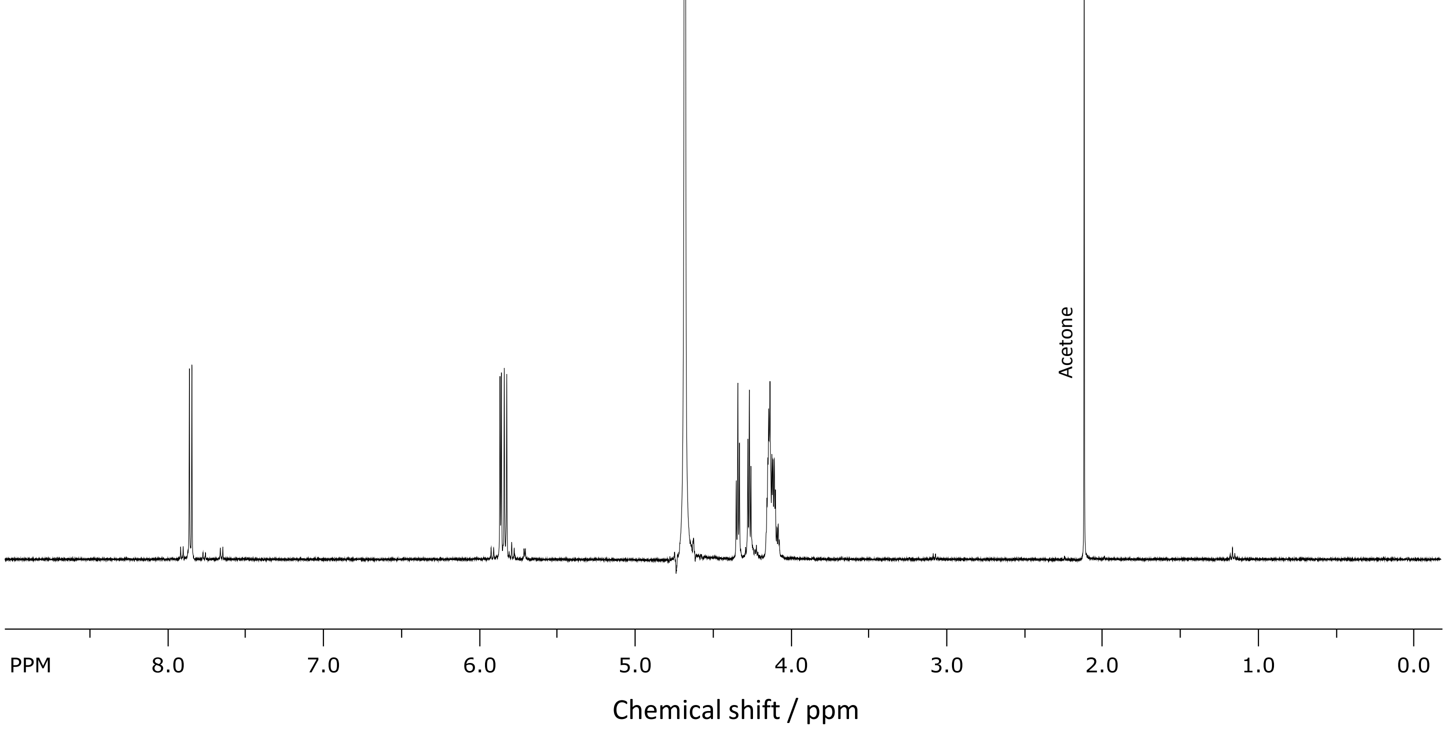

^{31}P NMR of 2´-deoxyadenosine 5´-diphosphate (2c)

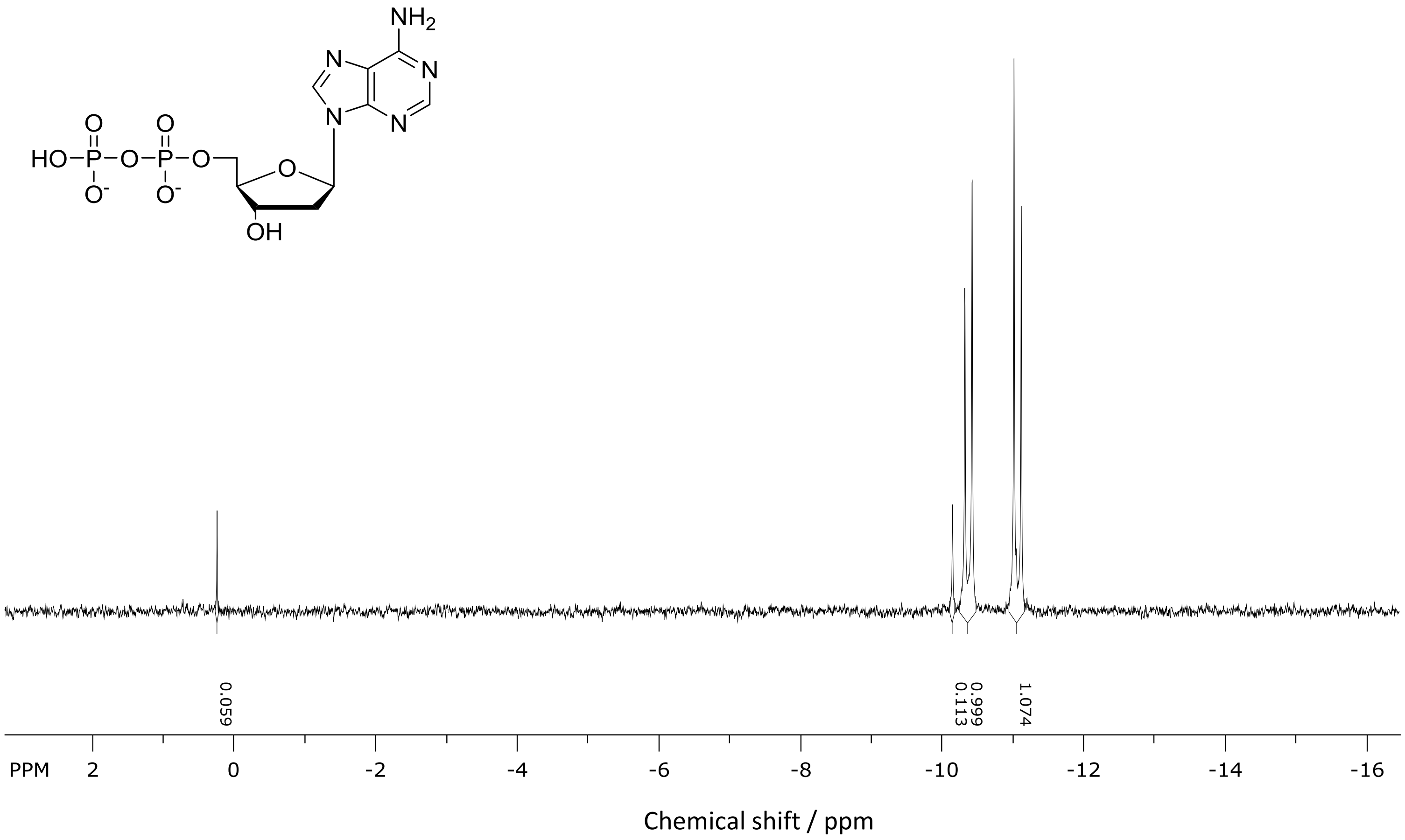

^{1}H NMR of 2´-deoxyadenosine 5´-diphosphate (2c)

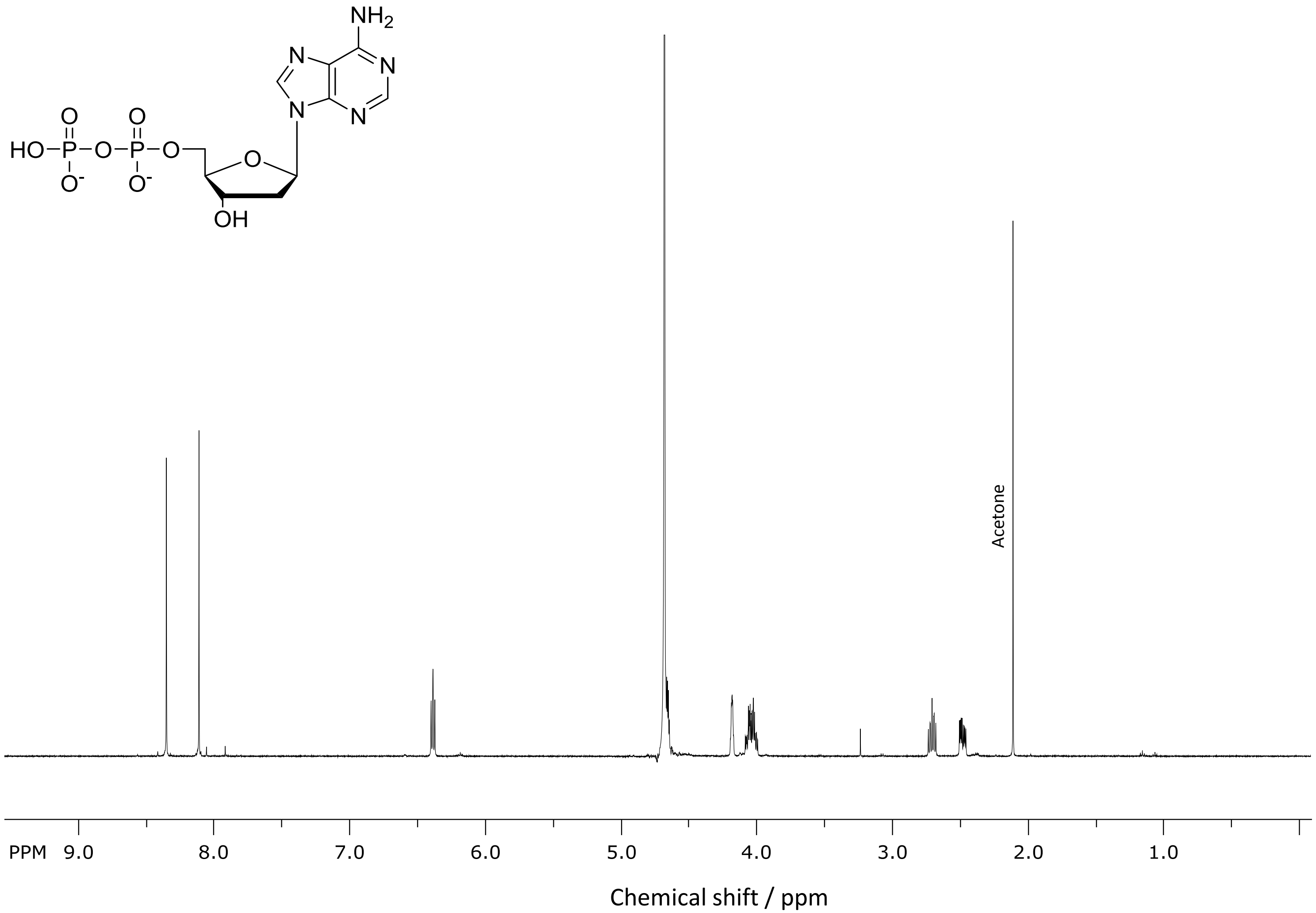

^{31}P NMR of adenosine 5´-diphosphate (2d)

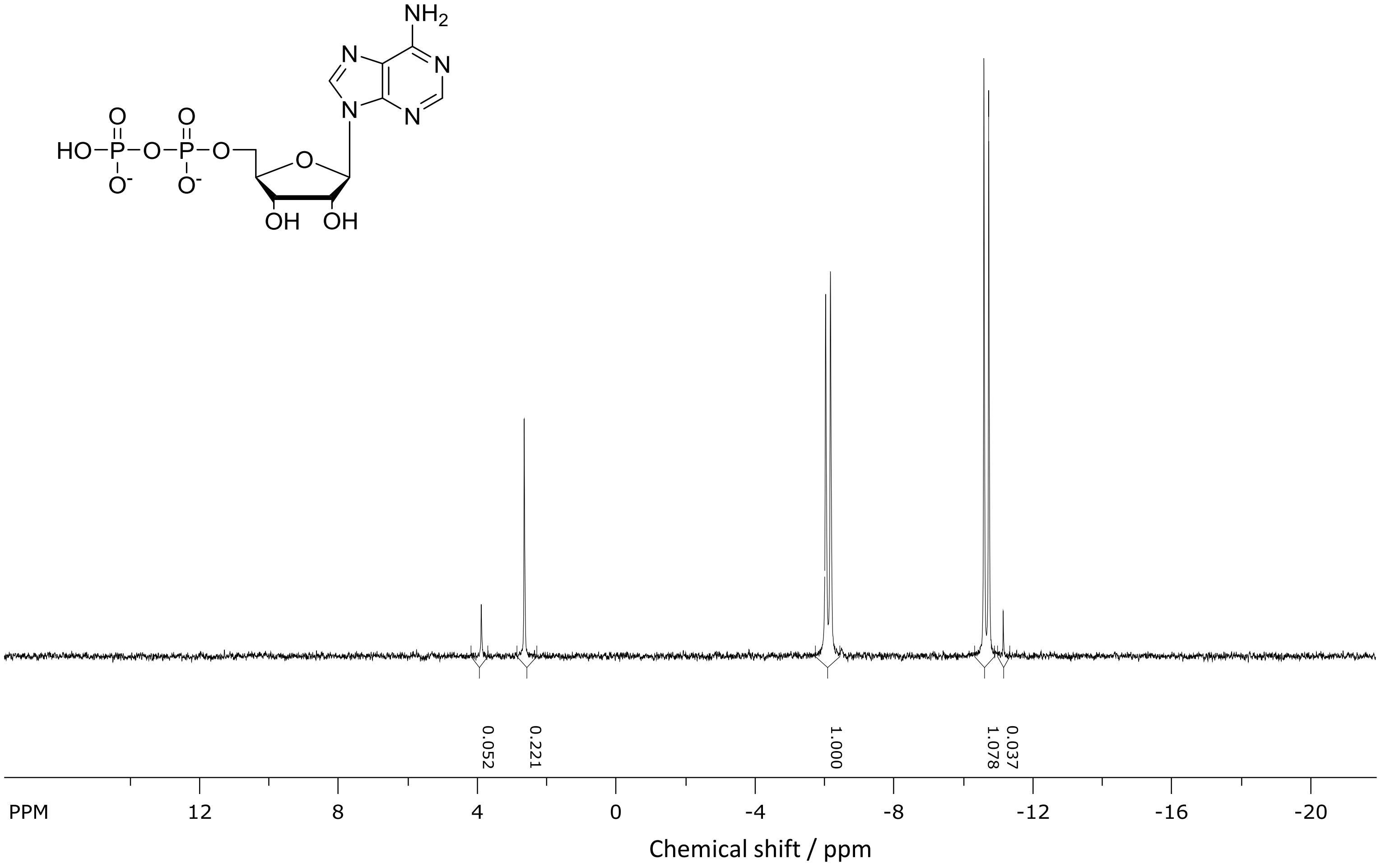

^{1}H NMR of adenosine 5´-diphosphate (2d)

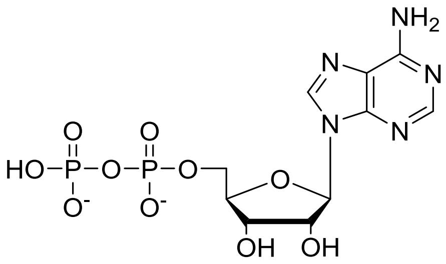

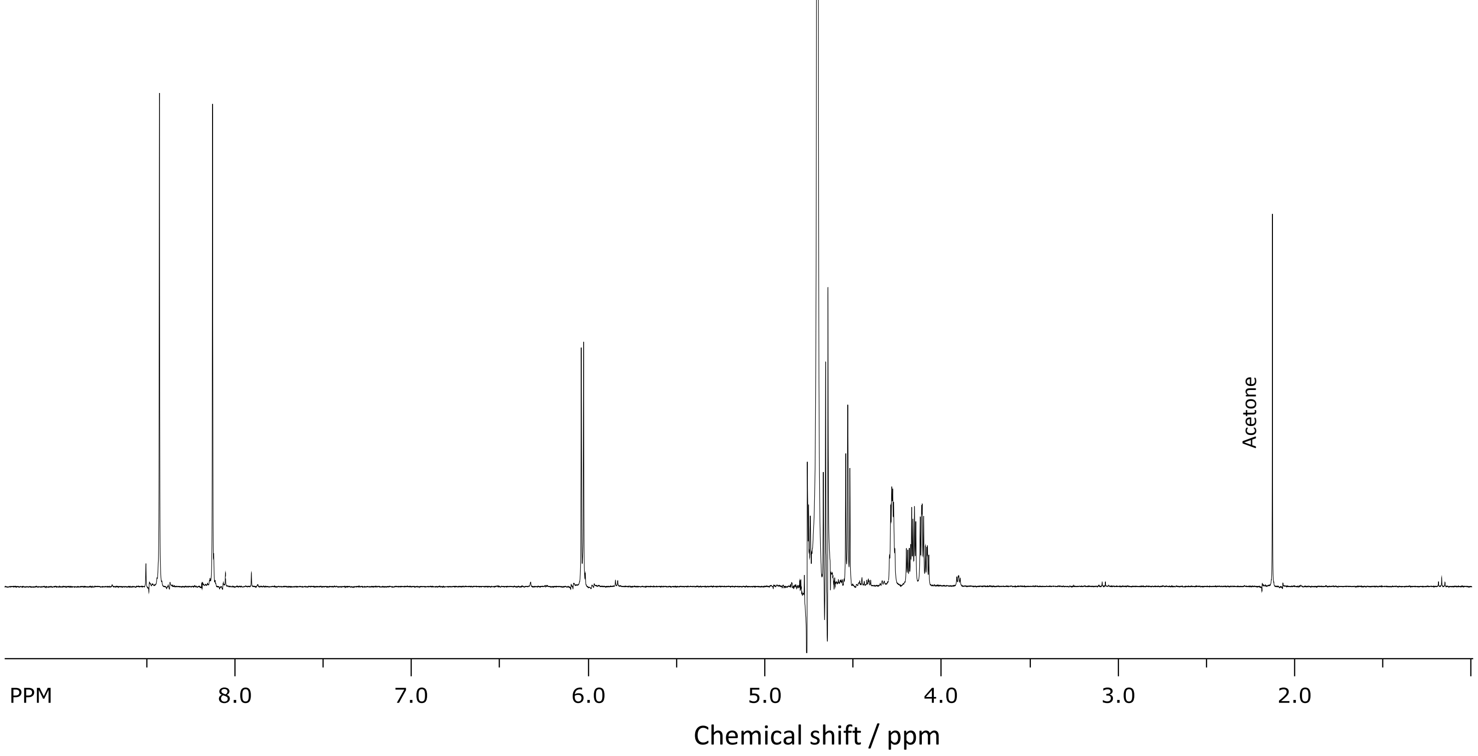

References

1. Davisson, V. J.; Davis, D. R.; Dixit, V. M.; Poulter, C. D. *J. Org. Chem.* **1987**, *52*, 1794-1801. doi:10.1021/jo00385a026

2. Prakash, G. K. S.; Zibinsky, M.; Upton, T. G.; Kashemirov, B. A.; McKenna, C. E.; Oertell, K.; Goodman, M. F.; Batra, V. K.; Pedersen, L. C.; Beard, W. A.; Shock, D. D.; Wilson, S. H.; Olah, G. A. *Proc. Natl. Acad. Sci. U.S.A.* **2010**, *107*, 15693-15698. doi:10.1073/Pnas.1007430107

3. Winans, K. A.; Bertozzi, C. R. *Chem. Biol.* **2002**, *9*, 113-129. doi:10.1016/S1074-5521(02)00093-5

4. Wang, R.; Steensma, D. H.; Takaoka, Y.; Yun, J. W.; Kajimoto, T.; Wong, C. H. *Bioorg. Med. Chem.* **1997**, *5*, 661-672. doi:10.1016/S0968-0896(97)00005-9
